# Hydrogen sulfide maintains dental pulp stem cell function via TRPV1-mediated calcium influx

**DOI:** 10.1038/s41420-018-0071-4

**Published:** 2018-06-27

**Authors:** Ruili Yang, Yi Liu, Tingting Yu, Dawei Liu, Songtao Shi, Yongsheng Zhou, Yanheng Zhou

**Affiliations:** 1 Department of Orthodontics, Peking University School and Hospital of Stomatology, 100081 Beijing, China; 2 National Engineering Laboratory for Digital and Material Technology of Stomatology, Beijing Key Laboratory of Digital Stomatology, 100081 Beijing, China; 3 Department of Anatomy and Cell Biology, School of Dental Medicine, University of Pennsylvania, Philadelphia, PA 19104 USA; 4 Laboratory of Tissue Regeneration and Immunology and Department of Periodontics, Beijing Key Laboratory of Tooth Regeneration and Function Reconstruction, Capital Medical University School of Stomatology, 100050 Beijing, China; 5 Department of Prosthodontics, Peking University School and Hospital of Stomatology, 100081 Beijing, China

**Keywords:** Stem cells, Cell signalling

## Abstract

Hydrogen sulfide (H_2_S), an endogenous gasotransmitter, mediated a variety of biological processes through multiple signaling pathways, and aberrant H_2_S metabolism has been associated with mesenchymal stem cell (MSC) dysfunction. Here we employed the small interfering RNA treatment for cystathionine β-synthase (CBS), cystathionine γ-lyase, the main enzymes to synthesize H_2_S, and CBS-knockout mice to analyze the effect of H_2_S on dental pulp homeostasis. We showed that H_2_S deficiency attenuated dental pulp stem cell (DPSC) osteogenic/dentinogenic differentiation in vitro and in vivo with enhanced cell proliferation. Mechanically, H_2_S facilitated the transient receptor potential action channel subfamily V member 1-mediated calcium (Ca^2+)^ influx, which subsequently activated the β-catenin pathway. While H_2_S deficiency decreased Ca^2+^, resulting in glycogen synthase kinase-3β-mediated β-catenin degradation, which controls proliferation and differentiation of DPSCs. Consistently, H_2_S-deficient mice displayed disturbed pattern of dental pulp and less dentin formation. In this study, we identified a previously unknown mechanism by which H_2_S regulates DPSC lineage determination and dental pulp homeostasis.

## Introduction

Hydrogen sulfide (H_2_S) could freely diffuse through cell membranes to elicit various cellular events. H_2_S is synthesized by three enzymes, namely cystathionine β-synthase (CBS), cystathionine γ-lyase (CSE), and 3-mercaptopyruvate sulfurtransferase (MPST) from l-cysteine^1^. Alternations of H_2_S metabolism have been linked to osteoporosis and T cell-related immune disorders^2,3^. Endogenous H_2_S is essential to maintain differentiation and proliferation of neural stem cells and human-induced pluripotent stem cells, and to restore the function of endothelial progenitor cell and mesenchymal stem cells (MSCs) to ensure bone homeostasis^4,5^. Our previous studies showed that H_2_S deficiency resulted in bone marrow MSC impairment caused by H_2_S deficiency reduced sulfhydration of combined Ca^2+^ transient receptor potential (TRP)-mediated (transient receptor potential action channel subfamily V member 3 (TRPV3), TRPV6, and TRPM4) aberrant Ca^2+^ influx^6^. H_2_S donor was also reported to prevent trabecular bone loss induced by ovariectomy via increased Wnt ligands Wnt16, Wnt2b, Wnt6, and Wnt10b in the bone marrow^7^.

The oral cavity is abundant with a plethora of bacteria, some of which are well known to produce H_2_S. When the dynamic ecological equilibrium in the biofilm is disturbed, bacteria may elicit oral diseases such as caries, gingivitis, and periodontitis^8^. Several studies have indicated the role of H_2_S in dental stem cells^9^. Dental pulp stem cells (DPSCs), isolated from dental pulp, were reported to be highly proliferative and capable of differentiating into a variety of cells including odontoblasts, osteoblasts, adipocytes, and neural cells. DPSCs could be induced to generate bone and dentin and play an important role in maintaining dental pulp and dentin homeostasis^10^. Recently, studies showed that H_2_S increased DPSC hepatic differentiation^11,12^. Exogenous H_2_S induced DPSC apoptosis by activating mitochondrial pathway apoptosis^13^. H_2_S may also be involved in periodontal ligament stem cell proliferation and differentiation and orthodontic tooth movement^14–17^. However, it remains unclear as to how H_2_S molecularly maintains homeostasis of DPSCs and dental pulp.

In this study, we showed that the gasotransmitter H_2_S is essential to maintain DPSC function via TRPV1-mediated Ca^2+^ influx-stimulated glycogen synthase kinase-3β (GSK3β)/β-catenin pathway.

## Results

### DPSCs expressed CBS, CSE, and produced H_2_S

**Figure 1:**
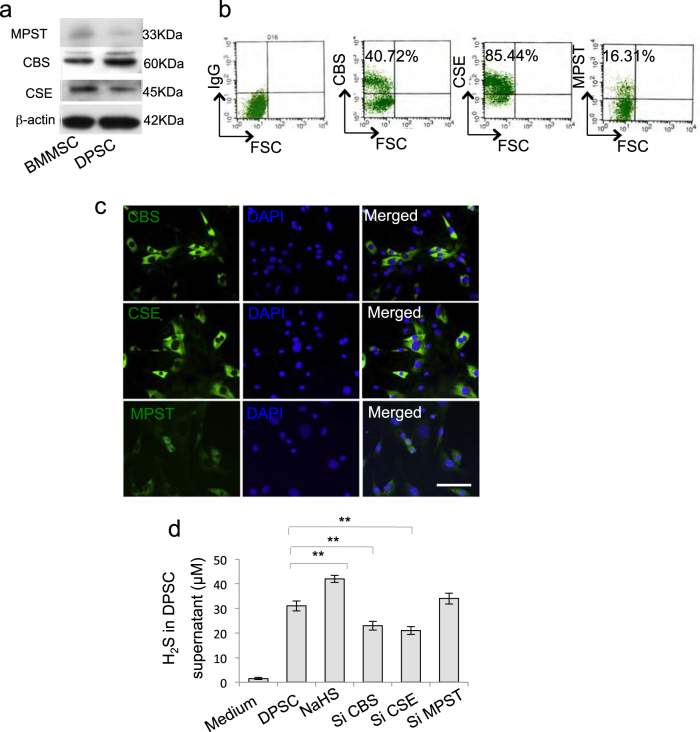
Human dental pulp stem cells (DPSCs) produced H_2_S. **a** Western blot analysis showed that DPSCs expressed CBS, CSE, and MPST as BMMSCs. **b**, **c** DPSCs expressed CBS, CSE, and MPST assessed by flow cytometry (**b**) and immunofluorescence staining (**c**). **d** DPSCs produced H_2_S in the culture supernatant. Knockdown of CBS or CSE, but not MPST, by siRNA decreased the production of H_2_S by DPSCs, while NaHS treatment could upregulate H_2_S concentration. **P < 0.01; scale bar: 20 μm. All experimental data were verified in at least three independent experiments

Aberrant H_2_S metabolism has been linked to MSC dysfunction; here we revealed that DPSCs expressed CBS, CSE, and ubiquitous enzyme MPST, the main catalyzed enzymes of H_2_S synthesis (Fig. 1a, c and Supplementary Figure 1). Meanwhile, DPSCs produced H_2_S in the culture supernatant, which was downregulated by CBS small interfering RNA (siRNA) or CSE siRNA treatment but not MPST siRNA treatment (Fig. 1d), and H_2_S concentration was upregulated by H_2_S donor NaHS treatment (Fig. 1d).

### H_2_S maintained DPSC function

**Figure 2:**
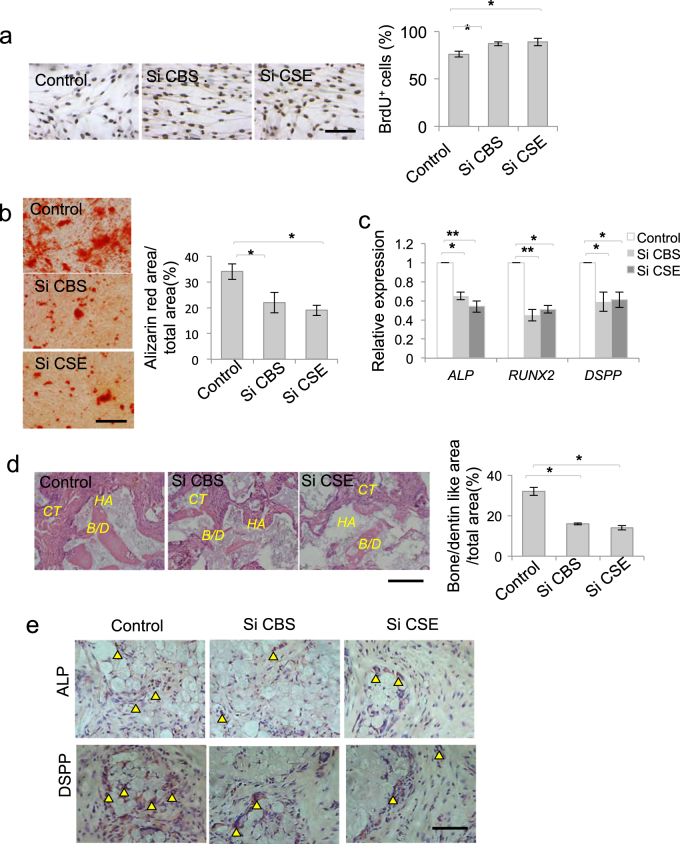
H_2_S was required to maintain homeostasis of DPSCs. **a** DPSC proliferation was increased after CBS or CSE siRNA treatment, as assessed by BrdU proliferation assay (**a**). **b**, **c** CBS or CSE siRNA treatment decreased DPSC osteogenic/dentiogenic differentiation, as assessed by Alizarin red staining, with decreased expression of *RUNX2*, *ALP*, and *DSPP* analyzed by qPCR. **d** When DPSCs were subcutaneously implanted into immunocompromised mice using HA/TCP as a carrier, bone/dentin-like hard tissue formation of DPSCs was decreased after CBS or CSE siRNA treatment. **e** ALP-positive and DSPP-positive cells were detected in the tissue formed by DPSC in vivo transplantation. CBS or CSE siRNA treatment decreased ALP-positive and DSPP-positive cells compared to the control ones. *P < 0.05, **P < 0.01; scale bar: 100 μm (**a**, **b**, **d**), 50 μm (**e**). HA HA/TCP, CT connect tissue, B/D bone/dentin. All experimental data were verified in at least three independent experiments

To analyze the role of H_2_S on DPSC function, we used CBS or CSE siRNA to treat DPSCs and found that DPSC proliferation was increased after CBS or CSE siRNA treatment (Fig. 2a and Supplementary Figure 2a). CBS or CSE siRNA treatment inhibited DPSC osteogenic/dentinogenic differentiation, accompanied by decreased expression of osteogenesis-related genes *RUNX2* and *ALP*, and dentin sialophosphoprotein (*DSPP*) (Fig. 2b, c). CSE, but not CBS, siRNA treatment decreased the adipogenic differentiation of DPSCs, along with decreased expression of adipogenesis-related genes *LPL* and *PPARγ* (Supplementary Figure 2b, 2c). When DPSCs were subcutaneously implanted into immunocompromised mice using hydroxyapatite/tricalcium phosphate (HA/TCP) as a carrier, bone/dentin-like hard tissue formation were observed. After CBS or CSE siRNA treatment, bone/dentin-like hard tissue formation of DPSCs was decreased (Fig. 2d). Moreover, ALP-positive and DSPP-positive cells were detected in the tissues formed by DPSC transplantation in vivo, while ALP-positive and DSPP-positive cells were decreased after CBS or CSE siRNA treatment (Fig. 2e). These data implied that DPSCs could differentiate into functional odontoblast-like cells and osteoblast in vitro and in vivo, which was attenuated by H_2_S deficiency.

### H_2_S activated TRPV1-mediated Ca^2+^ influx

**Figure 3:**
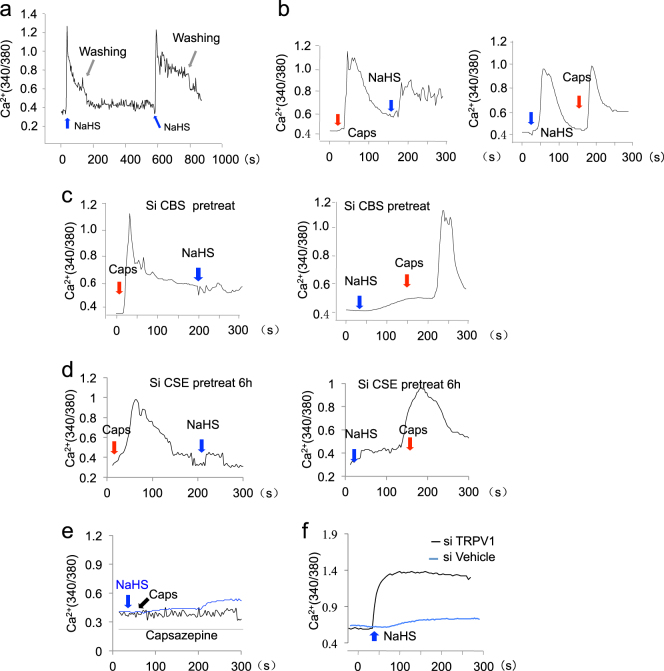
H_2_S activated TRPV1-mediated Ca^2+^ influx. **a** NaHS treatment could induce Ca^2+^ influx in DPSCs, as assessed by Ca^2+^ levels using the ratio of emission in response to excitation at 340 and 380 nm on an Olympus Optical IX71 microscope. **b** NaHS treatment could induce Ca^2+^ influx in DPSCs as capsaicin treatment. **c** Capsaicin, but not NaHS, could induce Ca^2+^ influx in CBS siRNA-pre-treated DPSCs. **d** Capsaicin, but not NaHS, could induce Ca^2+^ influx in CSE siRNA-pre-treated DPSCs. **e** Both capsaicin-induced and NaHS-induced Ca^2+^ influx was blocked by capsazepine treatment. **f** Knockdown of TRPV1 by siRNA could partially block the Ca^2+^ influx induced by NaHS treatment. Caps capsaicin. All experimental data were verified in at least three independent experiments

Emerging evidences showed that H_2_S plays crucial role in maintaining calcium homeostasis in endothelial cells, cardiomyocytes, and MSCs^18^. Here we found that H_2_S donor NaHS treatment induced Ca^2+^ influx in DPSCs (Fig. 3a). The TRPV subfamily is Ca^2+^-permeable cation channel, which could be activated by different stimuli. Our previous study revealed that combined TRPV3, TRPV6, and TRPM4 Ca^2+^ channels mediated Ca^2+^ influx-promoted osteogenic differentiation of BMMSCs^6^. First, we used TRPV3, TRPV6, and TRPM4 siRNAs individually or in combination to treat DPSCs and found that inhibition of these Ca^2+^ channels could not block NaHS-induced Ca^2+^ influx (Supplementary Figure 3). Studies found that TRPV1 was expressed in osteoblasts and was important in maintaining osteoblast differentiation ability^19^. Here we revealed that capsaicin, which acts on the TRPV1 receptor in nociceptive neurons to activate calcium influx, induced Ca^2+^ influx in DPSCs. NaHS treatment induced similar Ca^2+^ influx as capsaicin in DPSCs (Fig. 3b). When DPSCs were pretreated with CBS or CSE siRNA treatment, attenuated NaHS, but not capsaicin, triggered Ca^2+^ influx (Fig. 3c, d). When DPSCs were pretreated with capsazepine, a competitive antagonist of TRPV1, the Ca^2+^ influx induced both by NaHS and capsaicin were blocked, which indicated that TRPV1 channels play a crucial role in mediated H_2_S-induced Ca^2+^ influx in DPSCs (Fig. 3e). Furthermore, we found that knockdown of TRPV1 by siRNA blocked NaHS-triggered Ca^2+^ influx in DPSCs (Fig. 3f), verifying that H_2_S stimulates TRPV1 channel to trigger Ca^2+^ influx in DPSCs.

### TRPV1-mediated Ca^2+^ influx was required to maintain DPSC function

**Figure 4:**
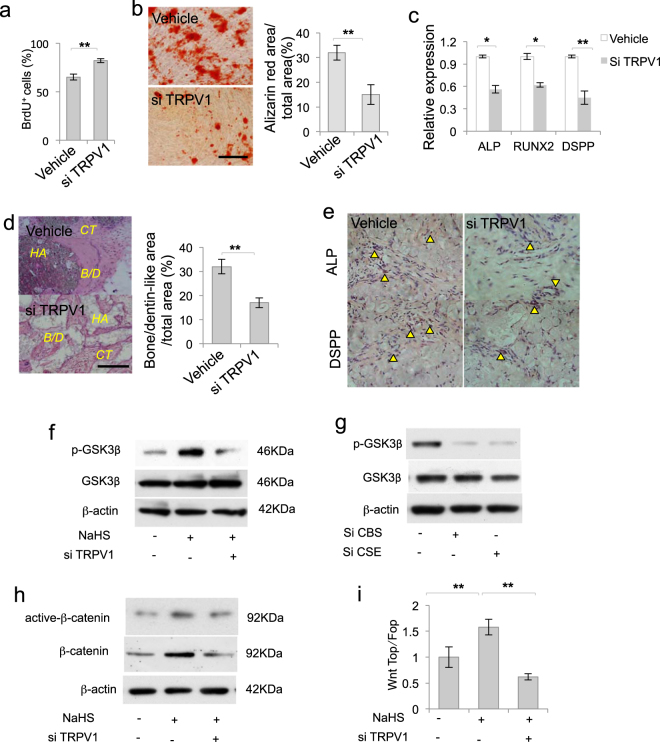
TRPV1-mediated Ca^2+^ influx was required to maintain DPSC function. **a** DPSC proliferation was increased after TRPV1 knockdown, as assessed by BrdU proliferation assay. **b**, **c** DPSC osteogenic/dentinogenic differentiation was decreased after TRPV1 knockdown, as assessed by Alizarin red staining, with decreased expression of *RUNX2*, *ALP*, and *DSPP* analyzed by qPCR. **d** Bone/dentin-like hard tissue formation of DPSCs in vivo decreased after TRPV1 knockdown by siRNA. **e** ALP-positive and DSPP-positive cells detected in tissues formed by DPSC transplantation were decreased after TRPV1 knockdown. **f** NaHS treatment upregulated p-GSK3β expression, and knockdown of TRPV1 by siRNA partially blocked NaHS-induced GSK3β phosphorylation. **g** CBS or CSE siRNA treatment downregulated p-GSK3β expression. **h** H_2_S donor NaHS treatment activated β-catenin expression, which was partially blocked after TRPV1 knockdown. **i** NaHS treatment enhanced TOPflash activity in DPSCs, which was blocked after TRPV1 siRNA treatment, as assessed by luciferase activity. NaHS treatment failed to alter FOPflash activity. HA HA/TCP, CT connect tissue, B/D bone/dentin. *P < 0.05, **P < 0.01; scale bar: 100 μm (**b**, **d**), 50 μm (**e**). All experimental data were verified in at least three independent experiments

To verify the role of TRPV1-mediated Ca^2+^ influx in DPSCs, we analyzed DPSC function after TRPV1 knockdown and revealed that DPSC proliferation was increased after TRPV1 knockdown (Fig. 4a). DPSC osteogenic/dentinogenic differentiation was decreased, along with downregulated expression of *RUNX2, ALP*, and *DPSS* after TRPV1 knockdown by siRNA (Fig. 4b, c). Moreover the in vivo bone/dentin-like hard tissue formed by DPSC transplantation also decreased after TRPV1 knockdown, which was consistent with H_2_S deficiency. (Fig. 4d). Consistently, less ALP-positive and DSPP-positive cells were detected in the tissues formed by DPSC transplantation after TRPV1 siRNA treatment (Fig. 4e). These studies indicate that TRPV1-mediated Ca^2+^ influx play crucial roles in maintaining DPSC function.

### H_2_S activated β-catenin signaling

Next, we analyzed the underlying mechanisms of how Ca^2+^ influx regulated DPSC function. We used H_2_S donor NaHS to treat DPSCs and found that NaHS treatment elevated phosphorylation of GSK3β, while knockdown of TRPV1 by siRNA blocked NaHS-induced phosphorylated-GSK3β (p-GSK3β) elevation (Fig. 4f). Consistently, we revealed that CBS or CSE siRNA treatment downregulated p-GSK3β expression (Fig. 4g). β-Catenin signaling plays crucial roles in determining stem cell lineage differentiation^1^. We found that H_2_S donor NaHS treatment increased β-catenin and active-β-catenin expression, which was blocked by TRPV1 knockdown (Fig. 4h). Next, we investigated whether H_2_S affected β-catenin transcriptional activity by using luciferase assay analyzed by the ratio of TOPflash and FOPflash. The results showed that H_2_S donor NaHS treatment increased pathway activity. TRPV1 siRNA treatment attenuated NaHS-induced pathway activity elevation (Fig. 4i). These data implied that H_2_S gasotransmitter pathway activated β-catenin signaling in DPSCs.

### H_2_S-mediated β-catenin signaling contributed to dentin formation

**Figure 5:**
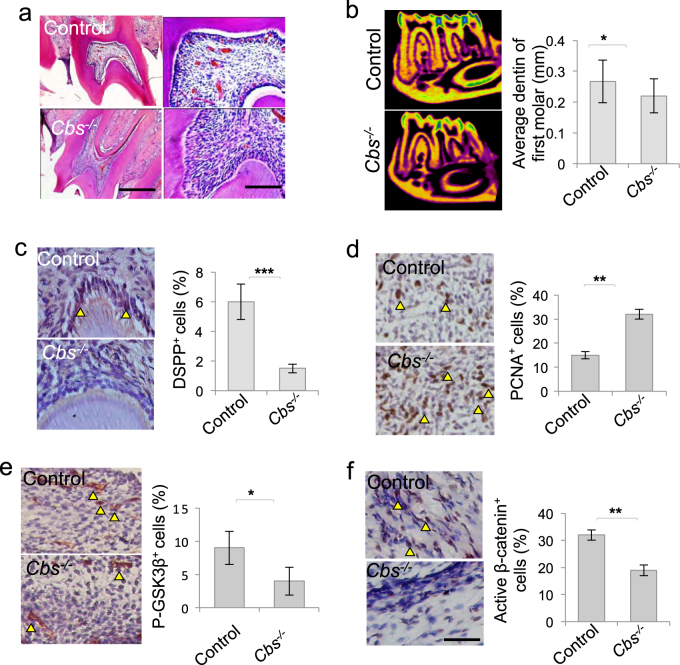
β-Catenin signaling were decreased in *Cbs*^−/−^ dental pulp. **a*** Cbs*^−/−^ mice dental pulp showed more cells than control ones. **b** The average dentin thickness of mandible first molar is less in *Cbs*^−/−^ mice than control ones, as assessed by micro-CT. **c** DSPP^+^ cells decreased in *Cbs*^−/−^ mice dental pulp compared to control ones. **d** PCNA^+^ cells increased in *Cbs*^−/−^ mice dental pulp. **e**, **f** p-GSK3β^+^ and active-β-catenin cells decreased in *Cbs*^−/−^ mice dental pulp. *P < 0.05, **P < 0.01, ***P < 0.001,   scale bar: 200 μm left panel, 100 μm right panel (**a**), 1 mm (**b**), 20 μm (**c**–**f**). All experimental data were verified in at least three independent experiments

In order to verify the role of β-catenin cascade in maintaining dental pulp homeostasis, we employed H_2_S-deficient (*Cbs*^−/−^) mice and found that *Cbs*^−/−^ mice dental pulp showed disturbed pattern with increased cell infiltration than control ones (Fig. 5a). The average thickness of dentin was decreased in *Cbs*^−/−^ mice with decreased DSPP-positive cells in the dental pulp compared to control ones (Fig. 5b, c). Furthermore, proliferative PCNA^+^ cells increased in *Cbs*^−/−^ mice dental pulp (Fig. 5d), while p-GSK3β^+^ and active-β-catenin^+^ cells decreased in *Cbs*^−/−^ mice dental pulp (Fig. 5e, f), which to some extent verifies that H_2_S-mediated β-catenin signaling is important in maintaining dentin formation and DPSC function in vivo.

## Discussion

H_2_S regulates several physiological and pathological processes via diverse mechanisms. In this study, we showed that H_2_S is essential to maintain DPSC osteogenic and dentinogenic differentiation. We used siRNA to knockdown CBS and CSE, the main enzymes that catalyzed H_2_S production in DPSCs, and found that DPSC proliferation was elevated. There were more PCNA^+^ cells infiltrated in *Cbs*^−/−^ mice dental pulp, indicating that H_2_S is required to restrict aberrant proliferation of stem cells, which is consistent with previous reports^15^. Furthermore, we found that the dentin thickness in *Cbs*^−/−^ mice decreased compared to the control ones, which was consistent with the decreased osteogenic and dentinogenic differentiation of H_2_S deficiency DPSCs in vitro and in vivo. These results expand the physiological roles of H_2_S in MSCs from bone to dental pulp and dentin homeostasis.

Calcium channels were one of main cellular events that may contribute H_2_S-mediated molecular responses^20,21^. The L-type and T-type Ca^2+^ channels and TRP channels are reported to be the alternative targets of H_2_S. TRP channels were involved in diverse cellular functions such as gene expression, proliferation, differentiation, migration, and apoptosis. It is reported that H_2_S triggers TRPV1 to mediate chloride secretion in acute pancreatitis and colon^22,23^. In this study, we found that H_2_S donors mimicked the effect of capsaicin in the neurons, leading to the Ca^2+^ flux entry in DPSCs. Ca^2+^ influx entry then triggered GSK3β/β-catenin cascade to regulate DPSC proliferation and differentiation. Furthermore, the study revealed that TRPV1 was essential to maintain DPSC proliferation and differentiation. When TRPV1 was knocked down by siRNA in DPSCs, the proliferation of DPSCs was elevated and osteogenic/dentinogenic differentiation of DPSCs was inhibited, which firstly implied the role of TRPV1 in maintaining MSC capacity. Our previous study reported that H_2_S-triggered Ca^2+^ influx is required to maintain bone and MSC homeostasis. The study revealed that combined TRPV3, TRPV6, and TRPM4 Ca^2+^ channels mediated Ca^2+^ influx to promote osteogenic differentiation of BMMSCs^6^. In this study, we found that the proliferation and differentiation capacity of DPSCs could not be attenuated by TRPV3, TRPV6, and TRPM4 Ca^2+^ channels blockage. These differences may attribute that DPSCs were derived from the neural crest and mesoderm during development, which were more accessible to differentiate into dentin-like tissue rather than the bone compared to BMMSCs. Functionally, DPSCs also easily differentiated into neuronal and glial cells and acted as neuronal cells^24,25^. These studies shed light on the different molecular target induced by H_2_S to illustrate the unique properties of MSCs derived from different tissues

Variety of signal pathways may contribute to that H_2_S fine-tune its effects on different tissues and cells. H_2_S may trigger the nuclear translocation of nuclear factor-κB (NF-κB) and affect the activity of numerous kinases including p38 mitogen-activated protein kinase, extracellular signal-regulated kinase, and Akt signaling. H_2_S is able to promote angiogenesis and vascular remodeling via the PI3K/Akt/survivin axis in endothelial cells by augmenting phosphorylation of ERK and p38^20,21^. H_2_S deceases pro-inflammatory genes involved in cardiac ischemic/reperfusion injury via inhibiting the nuclear translocation of NF-κB^26^. In BMMSCs, H_2_S-induced Ca^2+^ influx promoted β-catenin activity via the PKC/ERK pathway to maintain bone homeostasis^6^. However, we found that H_2_S treatment failed to alter ERK signaling in DPSCs (data were not shown). We showed that TRPV1-mediated Ca^2+^ influx entry facilitates the phosphorylation of GSK3β to disrupt the “destruction complex.” β-Catenin accumulates in the cytoplasm and then translocates into the nucleus, which promoted DPSC osteogenic differentiation. H_2_S deficiency decreased TRPV1-mediated Ca^2+^ influx entry, leading to decreased phosphorylation of GSK3β. These data indicated that the response of different stem cells to H_2_S may be regulated by different signaling pathways.

Cytoplasmic β-catenin was degraded, resulting in DPSC osteogenic differentiation deficiency. In vivo, Cbs^−/−^ mice dental pulp showed elevated cell infiltration and proliferation and decreased GSK3β/β-catenin signaling. Moreover, the dentin formation of *Cbs*^−/−^ mice was less than control ones. These data suggest that H_2_S-mediated GSK3β/β-catenin cascade is important to maintain DPSC function and dental pulp homeostasis. These results shed light on the different biology and mechanism of H_2_S targeting on different MSCs and tissue homeostasis.

## Conclusion

Our study reveals that the levels of H_2_S are essential to maintain Ca^2+^ homeostasis via TRPV1 Ca^2+^ channel in DPSCs. H_2_S deficiency results in Ca^2+^ influx/GSK3β/β-catenin cascade response in DPSCs, leading to differentiation deficiency of DPSCs and dentin formation disorders (Supplementary Figure 4).

## Materials and methods

### Mice

C57BL/6 (JAX #000664), B6.129P2-Cbstm1Unc/J (JAX #002461) *Cbs*^*+/****−***^ mice were purchased from Jackson lab. Female immunocompromised mice (beige nude/nude XIDIII) were purchased from Harlan Laboratories. All animal experiments were performed under institutionally approved protocols for the use of animal research at the University of Pennsylvania (IACUC# 805478) and Peking University (#LA2012-65).

### Antibodies and chemicals

#### Antibodies

Anti-active-β-catenin and β-catenin antibodies were purchased from Millipore (Temecula, CA, USA). Anti-CD105-PE, anti-CD146-PE, anti-CD90-PE, anti-CD34-PE, and anti-CD45-PE antibodies were purchased from BD Bioscience (San Jose, CA, USA). Unconjugated anti-GSK3β and anti-phospho-GSK3β antibodies were purchased from Cell Signaling Inc. (San Francisco, CA, USA). Anti-β-actin antibody was purchased from Sigma-Aldrich Corporation (St. Louis, MO, USA). Unconjugated anti-CBS, CSE, DSPP (DSP)  and TRPV1 were purchased from Abcam Inc. (Cambridge, MA, USA).

#### Chemicals

siRNA for CBS (hydroxylamine), CSE (dl-propargylglycine), TRPV1, TRPV3, TRPV6, and TRPM4 were purchased from Santa Cruz. H_2_S donor NaHS was purchased from Sigma-Aldrich Corporation (St. Louis, MO, USA). Capsaicin and capsazepine were purchased from EMD Millipore (Billerica, MA, USA). The concentrations of capsaicin and capsazepine used for the treatment were 1 μM.

### Isolation and culture of human bone DPSCs

Dental pulp from human extracted wisdom teeth were gently separated, minced, and digested with 2 mg/ml collagenase type I (Worthington Biochemical, Freehold, NJ, USA) and 4 mg/ml dispase II (Roche Diagnostics, Indianapolis, IN, USA) in phosphate-buffered saline for 1 h at 37 °C. Then, the cells were passed through a 70-μm strainer (BD Biosciences, Franklin Lakes, NJ, USA) to get single cells. The single-cell suspensions were cultured with α-minimum essential medium (α-MEM) supplemented with 15% fetal bovine serum (FBS), 100 mM l-ascorbic acid-2-phosphate, 2 mM l-glutamine, 100 U/ml penicillin, and 100 μg/ml streptomycin and passaged, as previously reported^27^.

### Human bone marrow MSCs BMMSC culture

Human bone marrow aspirates from healthy adult donors (20–35 years of age) were purchased from AllCells (Emeryville, CA, USA). Human bone marrow progenitor cells were enriched using fresh human bone marrow by RosetteSep^®^ Human Bone Marrow Progenitor Cell Pre-Enrichment Cocktail (STEMCELL Technologies, Vancouver, BC, Canada) following the manufacturer’s instructions. The enriched single-cell suspensions were cultured with α-MEM supplemented with 15% FBS, 100 mM l-ascorbic acid-2-phosphate, 2 mM l-glutamine, 100 U/ml penicillin, and 100 μg/ml streptomycin and passaged, as previously reported^27^.

### Cell proliferation assay

DPSCs were passaged to a 4-well chamber slide (Nunc, 1 × 10^4^ per well) and incubated for 12 h at 37 °C in a 5% CO_2_ condition. Then, the cells were assayed for bromodeoxyuridine (BrdU) incorporation, as per the manufacturer’s instruction. BrdU^+^ and total cell numbers were counted in five images per subject. The BrdU^+^ cells were indicated as a percentage of BrdU^+^ cells and the total cell number.

### DPSC-mediated hard tissue formation in vivo

Approximately 4.0 × 10^6^ DPSCs were mixed with HA/TCP ceramic particles (40 mg, Zimmer Inc., Warsaw, IN, USA) as a carrier and subcutaneously implanted into the dorsal surface of 8- to 10-week-old immunocompromised mice (n = 4). At 8 weeks post-implantation, the implants were harvested, fixed in 4% paraformaldehyde, and then decalcified with 5% EDTA, followed by paraffin embedding. The 5 μm paraffin sections were stained with hematoxylin and eosin and analyzed by NIH ImageJ. The newly formed mineralized tissue area in each field was calculated and shown as a percentage of the total tissue area.

### Osteogenic/dentinogenic differentiation assay

Osteogenic/dentinogenic differentiation is performed as described in the Supplementary materials.

### Adipogenic differentiation assay

Adipogenic differentiation is performed as described in the Supplementary materials.

### Immunofluorescent staining

DPSCs were seeded on a 4-well chamber slide and incubated for 12 h at 37 °C in a 5% CO_2_ condition. The detailed method of immunofluorescent staining is described in the Supplementary materials.

### Immunohistochemical staining

Immunohistochemical staining is performed as described in the Supplementary materials.

### Western blot

Western blot is performed as described in the Supplementary materials.

### Quantitative PCR

For quantitative PCR (qPCR) analysis, total RNA was extracted with RNeasy Mini kit (Qiagen, Valencia, CA, USA), and complementary DNA was prepared using a SuperScript^®^ III Reverse Transcriptase (RT) kit (Invitrogen). qPCR was carried out using SYBR^®^ Green Supermix (Bio-Rad) on a Bio-Rad CFX96 Real Time System, as indicated by the manufacturer’s protocol.

### Measurement of Ca^2+^ image

DPSCs (1 × 10^5^) were seeded onto 60 mm culture dishes and cultured for 24 h at 37 °C in 5% CO_2_. Then, the cells were loaded with fura-2 AM (Invitrogen) and incubated for 1 h at 37 °C in 5% CO_2_ in the dark. Ca^2+^ levels were measured from the ratio of emission in response to excitation at 340 and 380 nm on an Olympus Optical IX71 microscope.

### Measurement of H_2_S

Cell culture supernatants were mixed with 0.25 ml Zn acetate (1%) and 0.45 ml water for 10 min at room temperature. TCA (10%; 0.25 μl) was then added and centrifuged (14,000 × *g* for 10 min at 4 °C). The supernatant was then collected and mixed with *N*,*N*-dimethyl-*p*-phenylenediamine sulfate (20 μM) in 1.2 M HCl and FeCl_3_ (30 μM) in 1.2 mol/l HCl. After 20 min, absorbance was measured at 650 nm.

### In vitro transcription assays

β-Catenin-mediated transcriptional activity was measured by the luciferase activity by using TOPflash and FOPflash described previously^6^. DPSCs (1 × 10^5^) were seeded onto a 24-well culture plate and cultured for 24 h. TOPflash or FOPflash (10 ng/well) and renilla luciferase (4 ng/well) were transfected into DPSCs using a Lipofectamine™ RNAiMAX kit (Invitrogen). After 15 h, the plates were assayed for firefly luciferase and renilla luciferase activities using the Dual-Luciferase^®^ Reporter Assay System (Promega).

### Micro computed tomography

The mandible of mice was scanned with a micro-computed tomography (micro-CT) system (Inveon MMCT, Berlin, Germany) at 80 kV, 500 µA, and an image voxel size of 18 µm. The hemi-mandibles were excised (*n* = 6 per group). The hemi-mandible samples were directed parallel to the occlusal plane and scanned for 2.70 mm. The thickness of the first molar was measured with the DataViewer software (ver. 1.4.3; SkyScan).

### Statistical analysis

*P* values were analyzed from two-tailed Student’s *t* test for the difference between two groups or one-way analysis of variance to compare the difference from more than two groups using the SPSS 18.0 software. *P* values <0.05 were considered significant.

## Supplementary information

supplementary material
