# Supplementary material for: Hydrogen sulfide maintains dental pulp stem cell function via TRPV1-mediated calcium influx
Source: Cell Death Discov. 2018 Jun 27;4:69. doi: 10.1038/s41420-018-0071-4 (PMC6060166; doi:10.1038/s41420-018-0071-4)
Supplement: Supplementary file 1 — supplementary material [file 41420_2018_71_MOESM1_ESM.pdf]

Supplementary

MATERIALS & METHODS

Osteogenic/dentinogenic differentiation assay.

DPSCs were cultured under osteogenic medium containing α-MEM supplemented with 15% FBS, 100 mM L-ascorbic acid-2-phosphate, 2 mM L-glutamine, 100 U/ml penicillin, and 100 μg/ml streptomycin, 1.8 mM KH_2PO4 and 10 nM dexamethasone (Sigma–Aldrich). After 28 days of induction, the cultures were stained with Alizarin Red. Expression of the osteogenic/odontoblastic marker *ALP*, *RUNX2 and DSPP* was assayed by qPCR analysis. The primers used for qPCR analysis were listed in supplementary table.

Adipogenic differentiation assay.

DPSCs were cultured under adipogenic medium containing α-MEM supplemented with 15% FBS, 100 mM L-ascorbic acid-2-phosphate, 2 mM L-glutamine, 100 U/ml penicillin, and 100 μg/ml streptomycin, 0.5 mM Isobutylmethylxanthin (Sigma–Aldrich), 60 μM Indomethacin (Sigma–Aldrich), 0.5 μM Hydrocortisone (Sigma–Aldrich), and 10 μg/ml Insulin (Sigma–Aldrich). After 14 days of adipogenic induction, the cultures were stained with Oil Red O. Expression of the adipogenic lineage-specific genes *LPL* and *PPARγ* was assayed by qPCR. The primers used for qPCR analysis were listed in supplementary table.

Immunofluorescent staining.

DPSCs were seeded on a 4-well chamber slide and incubated for 12 hours under 37 °C at 5% CO_2 condition. Then the slides were fixed in 4% paraformaldehyde, followed by 0.01% Triton-100 treatment for 10 min. The slides were blocked with normal serum matched to secondary antibodies for 1 hour, followed by incubation with the specific or isotype-matched antibodies (1:100) overnight at 4°C. Then the slides were treated with Rhodamin/FITC-conjugated secondary antibodies (1:200, Jackson Immuno Research, West Grove, PA) for 1 hour under 20-25°C in dark and mounted by means of a VECTASHIELD® Mounting Medium containing 4'6-diamidino-2-phenylindole(DAPI) (Vector Laboratories, Burlingame, CA).

Immunohistochemical staining

Dental pulp tissue sections from WT and *Cbs*$^{-/-}$ mice were used. Immunohistochemistry was performed with a two-step detection kit (Zhongshan Golden Bridge Biotechnology, China) as manufacturer's instruction. Primary antibodies against rat β-catenin (1:100), active-β-catenin (1:100), p-AKT(1:100), p-GSK3β (1:100) and CBS (1:200) were used. Positive and total cell numbers were counted in 5 images per subject. The positive cells (%) were indicated as a percentage of immunohistochemistry positive cells and the total cell number.

Western blot.

Cells were lysed in M-PER® mammalian protein extraction reagent (Pierce). Twenty µg total proteins were applied and separated on 4-12% NuPAGE® gel (Invitrogen) and transferred on Immobilon™-P membranes (Millipore

Corporation). The membranes were blocked with 5% non-fat dry milk and 0.1% Tween 20 for 1 h, followed by incubation with the primary antibodies (1:100-1000 dilution) at 4°C overnight. Then they were treated with horseradish peroxidase-conjugated rabbit or mouse IgG (Santa Cruz) (1:10,000) for 1 h, enhanced with a SuperSignal® West Pico Chemiluminescent Substrate (Pierce, Rockford, IL), and exposed on BIOMAX MR films (Kodak, Rochester, NY).

SUPPLEMENTARY FIGURE LEGEND

Supplementary Figure 1 (a) DPSCs expressed mesenchymal stem cell markers CD105, CD146 and CD90, but not hematopoietic markers CD34 and CD45, as assessed by flow cytometry, (b) The efficacy of CBS and CSE siRNA knockdown. ** $P<0.01$. All experimental data were verified in at least three independent experiments.

Supplementary Figure 2 H_2S was required to maintain homeostasis of DPSCs. (a) CBS and CSE siRNA treatment did not alter DPSC cell viability. (b, c) CSE, but not CBS siRNA treatment downregulated DPSC adipogenic differentiation, as assessed by Oil red O staining, with decreased expression of *LPL* and *PPARγ* analyzed by qPCR. * $P<0.05$, scale bar: 100 μm. All experimental data were verified in at least three independent experiments.

Supplementary Figure 3 H_2S activated TRPV1 mediated-Ca^{2+} influx. (a) The efficacy of TRPV3, TRPV6 and TRPM4 siRNA knockdown. (b) NaHS induced Ca^{2+} influx could not be blocked by TRPV3, TRPV6 and TRPM4 siRNA individually or combination treatment. * $P<0.05$, ** $P<0.01$. All experimental data were verified in at least three independent experiments.

Supplementary Figure 4 Abstract graphic Schematic showing that hydrogen sulfide (H_2S) metabolism regulates DPSC function and dental pulp homeostasis *via* Ca^{2+} influx activated β-catenin signaling. Mechanically, H_2S trigged Ca^{2+} influx *via* TRPV1 channel to phosphorylate GSK3β, facilitating β-catenin nucleus translocation.

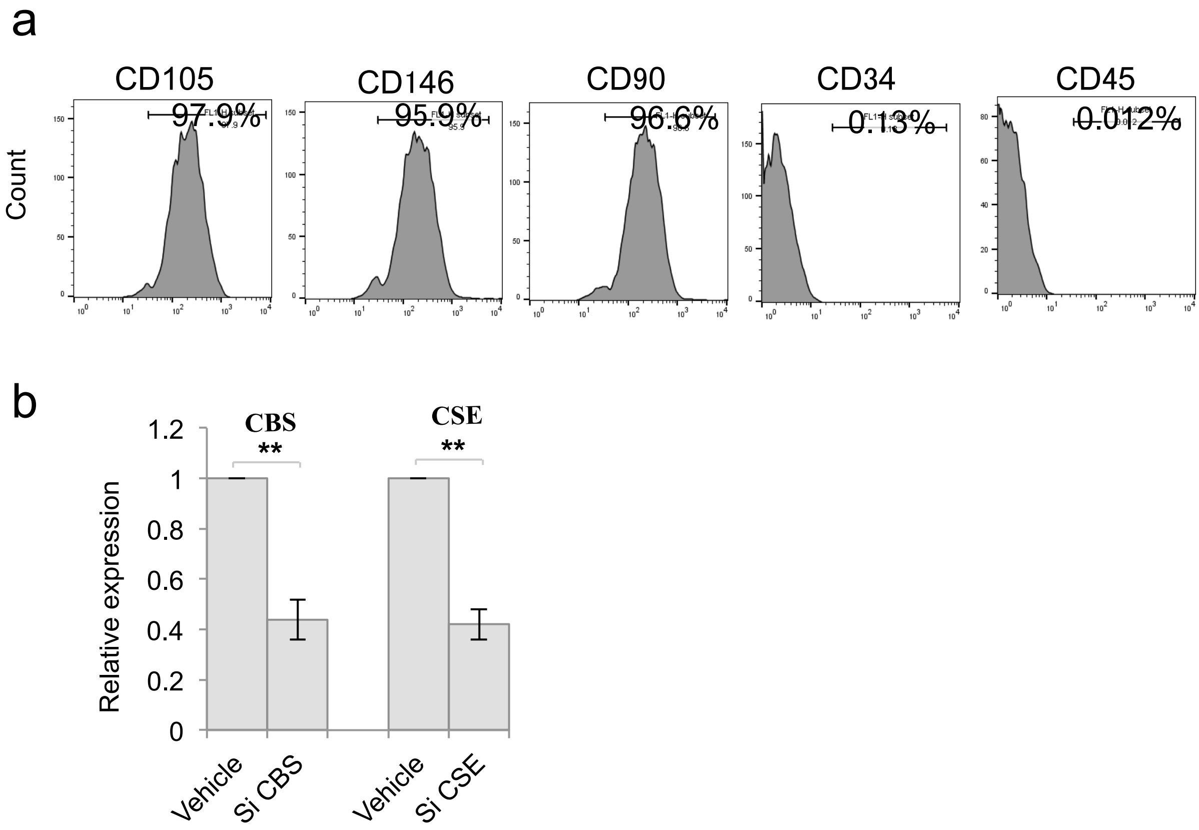

Supplementary Figure 1

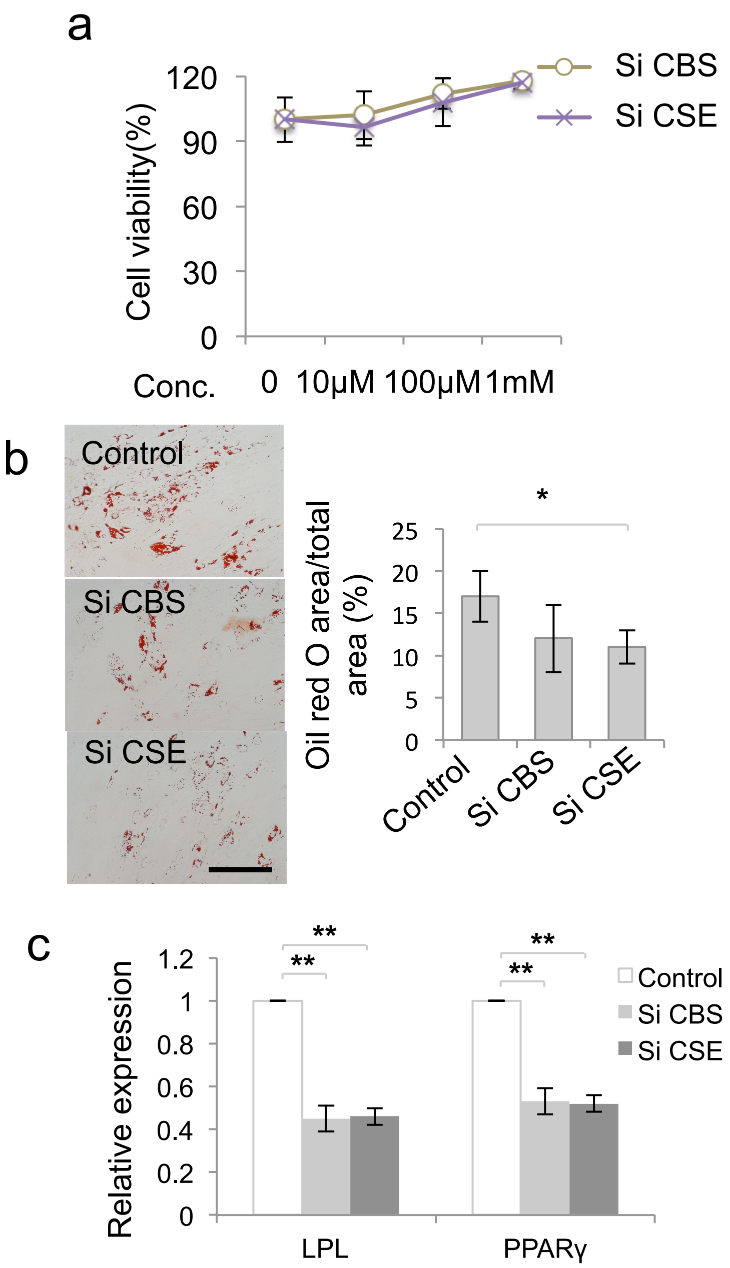

Supplementary Figure 2

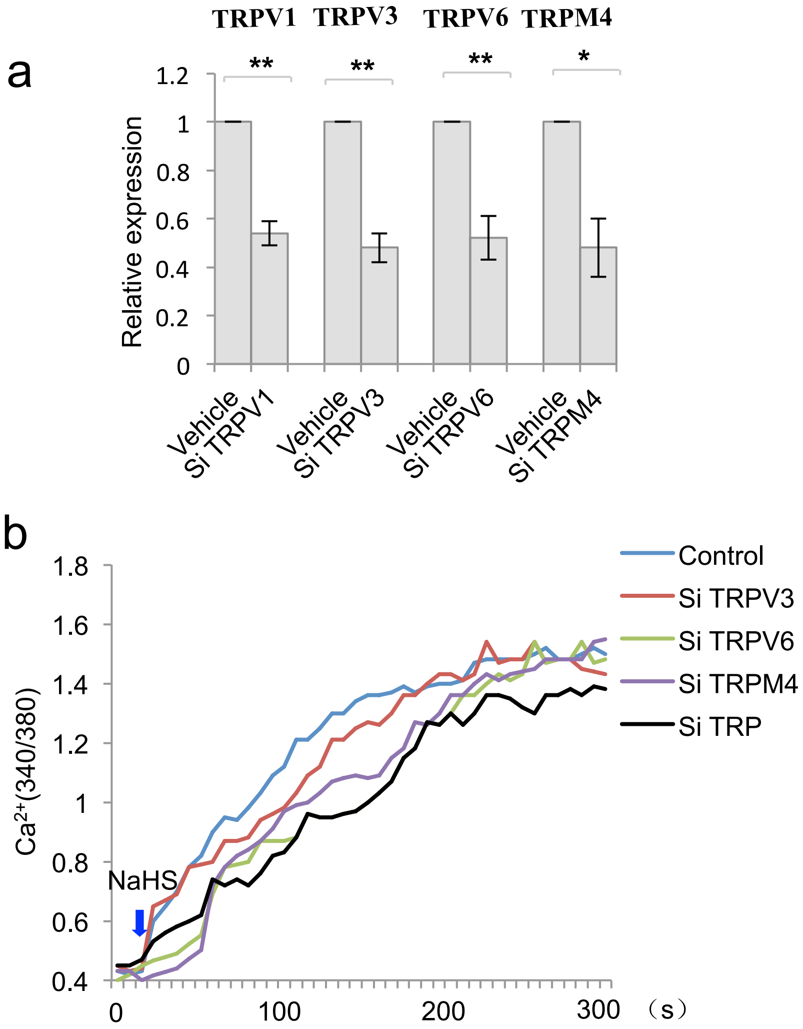

Supplementary Figure 3

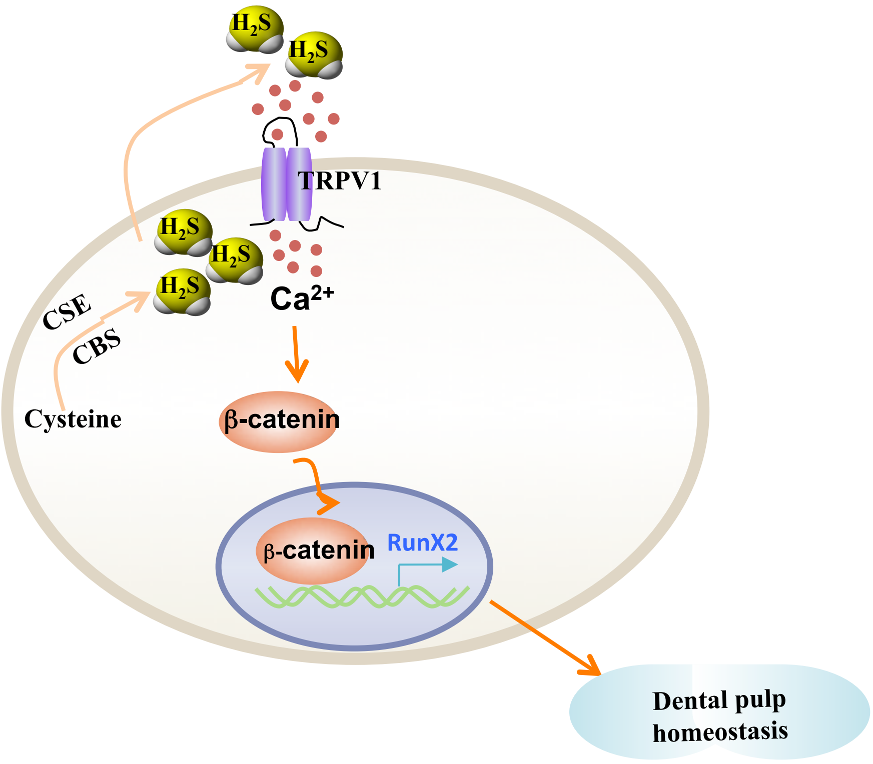

Supplementary Figure 4

Supplementary Table Primers used in this study

Gene	Strand	Sequence
ALP	F	GGAGTATGAGAGTGACGAGAAAG
	R	GAAGTGGGAGTGCTTGTATCT
RUNX2	F	CATCACTGTCCTTTGGGAGTAG
	R	ATGTCAAAGGCTGTCTGTAGG
DSPP	F	TCACAAGGGAGAAGGGAATG
	R	TGCCATTTGCTGTGATGTTT
LPL	F	GGACTGAGAGTGAAACCCATAC
	R	TGTGGAAACTTCAGGCAGAG
PPARγ	F	GCCTGCATCTCCACCTTATTA
	R	ATCTCCACAGACACGACATTC
